# Alcoholism: A Multi-Systemic Cellular Insult to Organs

**DOI:** 10.3390/ijerph15061083

**Published:** 2018-05-28

**Authors:** Ucee Dguzeh, Natasha C. Haddad, Kathia T. S. Smith, John O. Johnson, Angelia A. Doye, Judith K. Gwathmey, Georges E. Haddad

**Affiliations:** 1 Department of Physiology, New Jersey Medical School Rutgers, 185 S Orange Ave, Newark, NJ 07103, USA; Uceedguzeh@gmail.com; 2 Department of Physiology and Biophysics, College of Medicine, Howard University, 520 W Street, NW, #2309, Washington, DC 20059, USA; nmc.haddad@gmail.com (N.C.H.); kathia.smith@howard.edu (K.T.S.S.); jjohnson@howard.edu (J.O.J.); 3 Gwathmey Inc., Cambridge, MA 02138, USA; adoye@gwathmey.com (A.A.D.); jgwathmey@gwathmey.com (J.K.G.); 4 School of Medicine, Boston University, Boston, MA 02215, USA

**Keywords:** alcohol abuse, alcohol beneficial effects, multi-organ effects of alcohol

## Abstract

Alcohol abuse can affect more than the heart and the liver. Many observers often do not appreciate the complex and differing aspects of alcohol’s effects in pathophysiologies that have been reported in multiple organs. Chronic alcohol abuse is known to be associated with pathophysiological changes that often result in life-threatening clinical outcomes, e.g., breast and colon cancer, pancreatic disease, cirrhosis of the liver, diabetes, osteoporosis, arthritis, kidney disease, immune system dysfunction, hypertension, coronary artery disease, cardiomyopathy, and can be as far-reaching as to cause central nervous system disorders. In this review article, we will discuss the various organs impacted by alcohol abuse. The lack of clear guidelines on the amount and frequency of alcohol intake, complicated by personal demographics, make extrapolations to real-life practices at best difficult for public health policy-makers.

## 1. Introduction

In addition to motor vehicle deaths associated with alcohol intake, there is importantly a more chronic life-threatening impact of alcohol abuse on public health. Alcoholism is a pathology resulting from high alcohol intake [1]. Alcoholism is classified as an “Alcohol Use Disorder” (AUD)comprising several sub-categories, which refer, for instance, to a pattern of drinking which causes one to fail to meet everyday life obligations, interferes with the ability to make decisions or operate machinery, and engenders trouble legally and domestically. Another alcohol use disorder is alcohol dependence, which is diagnosed when there are clinically significant impairments. Criteria for such a diagnosis include (but are not limited to) a high level of alcohol tolerance, the presence of symptoms upon alcohol withdrawal, the rate of success in quitting, the time spent both drinking and recovering from its effects, and the failure to quit drinking for the sake of a separate physical or mental issue that is worsened by alcohol use. The Diagnostic and Statistical Manual of Mental Disorders (fifth edition) published by the American Psychiatric Association has made revisions regarding thresholds used for the diagnosis of alcohol use disorder (AUD) [2].

Alcoholism can progress from light drinking with little to no consequences to excessive uncontrollable drinking, which most often affects a person’s behavior. Persons with AUD most often lose the ability to regulate their drinking habits. Individuals who are struggling with alcohol abuse often feel they cannot function normally or reach their full potential without drinking alcohol. As a result, they often depend on it for psychological support. With time, chronic alcohol abuse can cause physical side effects and result in severe systemic complications.

Alcoholism is classified into three categories, i.e., moderate, binge, or severe. Moderate alcohol consumption is defined as one drink per day for women and two drinks per day for men. The National Institute on Alcohol Abuse and Alcoholism defines binge drinking as four drinks for women and five drinks for men within a 2 h period, which brings blood alcohol concentration above or equal to 0.08 g/100 mL. According to the Substance Abuse and Mental Health Services Administration, severe alcohol use corresponds to five or more days in a month of binge drinking. 

Despite the high incidence of alcohol use associated vehicular deaths, alcohol is also associated with more insidious disease processes that can impact multiple organs. Although the effects of alcohol on the development of systemic disease can differ depending on the person, severe complications of chronic drinking are often dependent on a person’s genetics, state of health, gender, body mass, and age, as well as the presence or absence of other co-morbidities. Therefore, chronic alcohol abuse can lead to multi-systemic diseases similar to what is experienced by diabetic patients.

**Figure 1:**
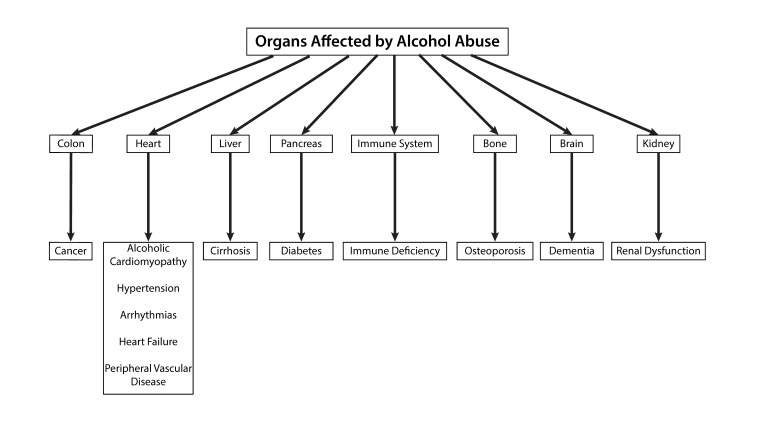
Known organs that show pathophysiological changes as a result of chronic alcohol abuse and the related clinical manifestations.

As shown in Figure 1, alcohol abuse is known to be associated with known pathophysiological changes [3] in most organs, e.g., breast cancer in women [4], colon cancer [5], pancreatic disease [6], cirrhosis of the liver [7], diabetes [8], osteoporosis [9], arthritis [10], kidney disease [11], gastrointestinal disorder [12,13,14], immune system dysfunction [15], hypertension [16], coronary artery disease [17], alcohol-induced cardiomyopathy [18], heart failure [19], and central nervous system disorders [20]. In this review article, we will discuss the multisystem effects of alcohol abuse and provide a cautionary tale about the benefits or lack thereof of alcohol consumption and related drinking patterns.

## 2. Systemic Effects of Alcohol

### 2.1. Cancer

Many studies have shown that drinking alcohol increases the risk of breast cancer. The risk of breast cancer increases by almost 7% for each alcohol drink consumed per day. Drinking 2–3 beverages per day leads to a 20% greater risk for breast cancer in women [4]. High estrogen levels have been reported to be associated with a higher risk for breast cancer, and one of the factors that increases estrogen levels is excessive alcohol consumption [4]. The World Health Organization International Agency for Research on Cancer (IARC) concluded that the risk of breast cancer increases with the increase of alcohol consumption, regardless of the beverage type.

Similarly, alcohol use has been linked to a high risk of cancer of the colon and rectum. Meta-analyses of large cohort studies as well as experimental studies suggest that chronic alcohol consumption increases the risk of gastric and colon cancer [5]. Ethanol is metabolized by alcohol dehydrogenases, catalase, or cytochrome to acetaldehyde, which is further oxidized to acetate by aldehyde dehydrogenase. Acetaldehyde is classified by the IARC as a group 1 carcinogen. The World Cancer Research Fund and the American Institute for Cancer Research have reported that consumption of more than 30 g/day of ethanol can be a cause of colorectal cancer.

### 2.2. Liver

When too much alcohol is consumed, it is not properly metabolized, and toxic metabolites accumulate and circulate in the body. The liver is the primary site of alcohol metabolism. Alcohol is detoxified and removed from the blood through a process called oxidation [21]. Oxidation prevents alcohol from accumulating in the cells and organs causing cell death. Women have lower amounts of the detoxifying enzymes, which is the reason why alcohol remains in women’s bloodstream longer than men’s.

There is an entire subcategory of diseases called alcohol-related liver diseases (ALD). The liver’s detoxifying abilities are used in part for toxic substances and can change toxins from harmful to beneficial or harmless, or eliminate them entirely from the body. According to the Germany Institute for Quality and Efficiency in Health Care, when alcohol is consumed in excess, liver cells die and are replaced by scar tissue. This phenomenon results in cirrhosis and is generally detected through blood tests, biopsy, Magnetic Resonnance Imaging (MRI), or ultrasound. Liver scarring as a result of cirrhosis makes it difficult for the liver to perform detoxification, leaving patients susceptible to inflammation and infection. For many patients suffering from cirrhosis, alcohol abstinence may ameliorate many of the associated clinical conditions. However, some individuals with severe liver cirrhosis will likely require a liver transplant [21].

### 2.3. Skeletal System

The skeletal system performs vital functions in addition to locomotion and biomechanical functions. The skeletal system is responsible in part for blood cell production, endocrine and immune regulation, and calcium storage [22]. Osteoporosis, a condition in which one’s bones become weakened as a consequence of bone density loss or vitamin D and calcium deficiencies, often manifests with alcohol-related liver disease (ALD). When alcohol is consumed excessively, it can inevitably lead to nutrient deficiency and loss of bone density. A study by the Korean National Health and Nutrition Examination Survey, in which participants self-reported their weekly alcohol intake and consented to tests for bone mineral density (BMD) by dual energy x-ray absorptiometry, showed that there was an inversely proportional relationship between alcohol intake and BMD. This finding corroborates the hypothesis that drinkers are at a significantly greater risk of osteoporosis.

### 2.4. Kidney Disease

All studies attempting to elucidate a direct link between alcohol abuse and kidney disease have been mostly inconclusive. However, there are some studies that offer hypotheses as to how alcohol abuse can engender kidney dysfunction [11]. One likely mechanism is through oxidative stress. Excessive consumption of alcohol causes an increased production of reactive oxygen species. The excess of free radicals causes tissue injury and inflammation, which have the capacity to cause tissue damage as well as cell death. In addition, the proven deteriorating effects of alcohol on other organs can contribute to damage of the kidneys [11].

### 2.5. Pancreas

Two important hormones produced by the pancreas are glucagon and insulin [6]. It is estimated that a person must engage in 10 to 15 years of heavy drinking (of at least 60 to 80 g of alcohol per day or about six to eight drinks per day), before pancreatitis becomes clinically apparent. The pancreas of persons with AUD often shows fibrosis, calcium deposits, and loss of functioning enzyme and hormone-producing cells, which may lead to poor digestion of food and loss of control over blood sugar levels. Other serious complications that may accompany AUD-associated pancreatitis are kidney failure, jaundice, and formation of pancreatitis pseudocysts. These cysts are harmful because, when they become too large, they may rupture in the abdominal cavity or cause hemorrhage by eroding into a large adjacent abdominal blood vessel [11].

Chronic complications in patients with AUD-associated pancreatitis arise as a result of inadequate pancreatic production of certain digestive enzymes and hormones. This is referred to as pancreatic insufficiency. The most common clinical outcomes are weight loss, steatorrhea, and diabetes mellitus. Diabetes mellitus results from the lack of insulin production by the pancreas. Its development denotes extensive loss of insulin-secreting cells within the atrophied pancreas. In addition, people consuming excessive amounts of alcohol can also suffer from acute pancreatitis [6]. Interestingly, moderate consumption of alcohol (<50 g/day for women and <60 g/day for men) has been demonstrated to be protective for type 2 diabetes, showing a U-shaped relationship in both gender [23].

### 2.6. Immune System

The immune system consists of a group of organs, cells, and tissues in the body that fight infection and disease. When people consume alcohol in excess, inflammation occurs at the cellular level and often activates the immune system. As a result, the body can become immuno-deficient [24]. Additionally, heavy drinking is associated with declining numbers of lymphocytes, resulting in higher susceptibly to bacterial and viral infections. In that regard, a recent study by Szabo’s group [25] showed that the remarkable activation and recruitment of inflammatory monocytes and macrophages associated with ALD is mediated by reduced anti-inflammatory markers and enhanced activation of pro-inflammatory molecules, such as TNF-α and interleukins, within hepatic extracellular vesicles. Nonetheless, Coleman and Crews (2018) [26] discuss the positive correlation between the activation of the innate immune system and the pathological progression of AUD. Interestingly, heavy alcohol exposure seems to suppress the immune responses, but moderate alcohol consumption seems to have beneficial effects on the immune system [27].

### 2.7. Cardiovascular System

Chronic alcohol consumption is known to contribute to the occurrence of cardiovascular diseases by negatively impacting the vasculature of the heart and the peripheral vasculature and exerting direct negative effects on the myocardium. It is known that alcohol abuse can lead to coronary artery-related heart disease [17], hypertension [16], myocardial infarction [19], cardiomyopathy [18]), and cerebral stroke [28]. The abuse of alcohol results in pathophysiological changes in the heart and vasculature structure, and in related functional changes. Chronic alcohol consumption can result in heart failure and cardiomyopathy, which is a structural enlargement of the heart, as well as in atrial fibrillation (an electrical dysrhythmia). An estimated 2.7–6.1 million people in the United States have atrial fibrillation (AF). With the aging of the U.S. population, this number is expected to increase. Atrial fibrillation often requires either surgical ablation and/or medical management with anti-arrhythmic drugs, anti-coagulants, and rate-rhythm control medications, e.g., beta-blockers. Atrial fibrillation can result in emboli and lead to cerebral strokes [29].

#### 2.7.1. Clinical Studies and Public Health

Alcohol consumption has been directly linked to the occurrence of atrial fibrillation. In a study on the risk of atrial fibrillation associated with alcohol consumption, 88,782 men and women were examined during 2003–2010 [29]. Information on incident cases of atrial fibrillation was obtained from a validated nationwide registry. The results proved that high alcohol consumption was associated with the risk of atrial fibrillation among men but not among women.

The respective hazard ratios among men who drank 28–35 and over 35 drinks/week, were 1.40 and 1.62 (95% confidence intervals of 1.09–1.80 and 1.27–2.05, respectively) when compared with men who consumed 1 drink/week. The study concluded that alcohol consumption was associated with a higher risk of atrial fibrillation in men. A recent meta-analysis concluded that a high level of alcohol exposure is associated with an elevated risk for atrial fibrillation (AF), while no risk was found for a low alcohol intake [30].

There are also reports of the beneficial effects of alcohol consumption on the cardiovascular system [31]. According to one study, drinkers consuming less than 30 g of alcohol/day without ever engaging in heavy drinking had a lower risk of developing coronary artery disease than those who did not drink at all [17]. Additionally, it was advised that women drink less than men, as the effects of drinking become detrimental to women’s health more rapidly than to men's health because of the differences in body composition, biochemical homeostasis and tolerance levels. A meta-analysis in the British Medical Journal comparing varying levels of alcohol consumption and specific cardiovascular conditions found that a light to moderate alcohol intake was associated with a 25–35% decline of a person’s risk for coronary heart disease. However, the cardioprotective effects of alcohol consumption were found to be dose-dependent, with 2.5 g–14 g/day of alcohol being beneficial, and >60 g/day significantly increasing the risk of stroke [32].

A large case-control study combining observations from 52 countries reported an association between alcohol consumption and the risk of myocardial infarction [33]. The study found that heavy drinking (six drinks or more) might increase the risk of acute myocardial infarction (MI) in the following 24 h after alcohol consumption, especially in the elderly. However, according to subsequent studies, patients who survive an MI might benefit from moderate alcohol consumption because of the decreased risk of all-cause and cardiovascular mortality [31]. In the case of stroke, studies showed a decreased risk of stroke associated with mild alcohol exposure (<15 g/day), while moderate drinkers showed no difference [31] compared to non-drinkers. Furthermore, it was reported that in older patients heavy alcohol increased their risk of heart failure and hypertension, whereas moderate alcohol drinking did not show negative effects on cardiovascular function [34]. Similarly, a meta-analysis and review by Briasoulis et al., 2102, indicated an increased risk of hypertension in men with heavy alcohol exposure, which was less obvious in moderate and low drinkers. However, the relationship between hypertension and alcohol exposure showed a J-shaped curve in women.

The prevalence of alcohol-induced cardiomyopathy is most likely underreported, as autopsy findings commonly reveal pathologic characteristics of the heart that yield no clinical symptoms or diagnostic clues other than family reporting [18]. The most effective treatment for alcohol-induced cardiomyopathy is the cessation of alcohol intake. Although high amounts of alcohol consumption can be detrimental to the heart, alcohol can help avoid coronary artery disease (CAD)—a condition in which plaque builds up in the arteries—when used in moderation. Eight hundred and forty-three male twins age 42–55 years who were casual drinkers, without coronary artery disease were observed over a period of years [17]. Their alcohol use was recorded, and, over the years, 129 of the men died from CAD and 219 died from other cardiovascular conditions. The results found that in the majority of cases, the twin who suffered from CAD had consumed less alcohol. The study concluded that moderate alcohol consumption is associated with a lower CAD mortality risk. The belief is that alcohol slows the process by which plaques form in the coronary arteries [17]. Furthermore, moderate alcohol intake has been shown to impact cholesterol levels, i.e., High-Density Lipoproteins (HDL) and Low-Density Lipoproteins (LDL). Alcohol intake may increase plasma HDL levels by altering either the synthesis or the clearance of HDL [35].

Wine consumption has similarly been shown to be cardioprotective [33]. The term French paradox describes the relatively low incidence of cardiovascular disease in the French population and the benefits of consuming red wine. After nearly 20 years, several studies have investigated the positive biological and clinical association of red wine consumption with cardiovascular disease and mortality. Light to moderate intake of red wine produces a kaleidoscope of potentially beneficial effects that target all phases of the atherosclerotic process, from atherogenesis to vessel occlusion (flow-mediated dilation, thrombosis). Red wine components, especially resveratrol and other polyphenolic compounds, may decrease oxidative stress, enhance cholesterol efflux from the vessel walls (by increasing the levels of high-density lipoprotein cholesterol) and inhibit lipoproteins oxidation. Light-to-moderate red wine consumption is also associated with a favorable genetic modulation of fibrinolytic proteins, ultimately increasing the surface-localized endothelial cell fibrinolysis. Conversely, chronic heavy alcohol consumption and binge drinking are associated with an increased risk of cardiovascular events.

#### 2.7.2. Cellular Changes

The heart has not only electrical components that can be impacted by alcohol consumption, causing AF and other dysrhythmias, but also a mechanical function. The pumping of the heart (excitation–contraction coupling) is responsible for the force generated to “pump blood”. When the cardiomyocytes develop impaired contraction (systolic) and relaxation (diastolic) because of chronic alcohol abuse, cardiac enlargement and reduced ejection fraction may ensue, causing AUD-associated cardiomyopathy. Alcohol has been shown to be involved in about 33% of all cases of dilated cardiomyopathy [18,36].

We have reported that the observed detrimental effects of acute alcohol exposure on the heart are mediated through the modulation of the survival pathway known as the PI3K/Akt signaling. In particular, acute exposure to low alcohol, such as in casual drinking, decreases Akt activity as a result of reduced oxidative cellular stress. On the other hand, acute exposure to high alcohol, such as in binge drinking, leads to the activation of Akt as a response to increased cellular oxidative stress. This latter effect can inhibit AMPK, leading to contractile dysfunction. Accordingly, we have shown thatacute low alcohol improves the cardiac function in vivo through enhanced contractility and stroke volume, while acute high alcohol levels induce the opposite effect in a PI3K/Akt-dependent manner.

Interestingly, we have found that the beneficial cardioprotective effect of chronic alcohol intake are PI3K/Akt-independent. The enhanced contractility is instead associated with the activation of Nrf-2 survival pathway [37]. However, the reduced contractility and compliance of hearts chronically exposed to high levels of alcohol is mediated by a reduction in PI3K/Akt activity and a simultaneous increase in oxidative stress. Accordingly, oxidative stress is associated with cell damage and apoptosis. Thus, during low alcohol exposure, oxidative stress is low, and, therefore, there is no activation of the anti-apoptotic Akt signaling. However, chronic exposure to high levels of alcohol induces oxidative stress and reduction in Akt gene expression and protein activation. This negatively shifts the homeostatic regulation of oxidative stress, leading to the known detrimental effects of chronic high alcohol exposure. Thus, chronic high alcohol-induced oxidative stress is not appropriately regulated by Akt.

Heightened AF occurrence associated with elevated alcohol intake could be due to an increase in triggered atrial calcium waves induced by L-type calcium channels [38]. Thus, improper calcium channel expression and activity may contribute to alcohol-induced cardiomyopathy. In this regard, it was suggested that the dysregulation of protein synthesis and autophagy contribute to the reduced contractility seen with high alcohol exposure [39].

### 2.8. Central Nervous System

The forebrain, the midbrain, and the hindbrain can be affected by high chronic alcohol consumption. However, each part of the brain can be affected differently by alcohol abuse. Alcohol abuse has well-recognized neurological and psychological side effects. Neonatal alcohol syndrome, seen in infants born to chronic alcohol abusers, results in impaired neonatal brain development as well as physical and mental disorders.

Alcohol is known to promote brain damage and mental disorders. The neuropsychological consequences of brain damage resulting from chronic alcohol abuse include a change in behavior, memory loss (especially short-term memory), amnesia, and atrophy (shrinkage of the cerebral cortex) [40]. The areas of the brain most susceptible to alcohol-induced damage are the limbic lobe, the diencephalon, and the basal forebrain.

According to the right hemisphere hypothesis, the right hemisphere of the brain is more susceptible to damage from alcohol abuse, which might explain the disproportionate impact of alcohol on nonverbal, visuospatial, and emotional functions, all of which are thought to be governed by the right hemisphere [40]. In one study, people with AUD, average drinkers, and non-drinkers were given images to describe and analyze in order to gauge their emotional intelligence and competence. According to the results, the persons with AUD reported emotions that were more intense than the persons without AUD, which suggested that alcohol disrupts emotional competence and emotional intelligence. Additionally, although the persons with AUD were significantly more depressed than the non-drinkers, this did not significantly affect the performance of the two groups.

Estimates of the incidence of Parkinson’s disease range from 7.9 to 19 per 1,000,000 person/year, and a prevalence of 57 to 230 per 100,000 populations [41]. Although genetics and environmental exposures are recognized as contributors to Parkinson’s disease, researchers have also begun to investigate the relationship between alcohol consumption and the occurrence of Parkinson’s disease [41]. According to a review article on alcohol consumption and Parkinson’s disease, a prospective study conducted in Finland found that persons who consumed more than 5 g of alcohol per day had an increased risk of developing Parkinson’s disease compared to non-drinkers. Another study from the U.S.A. found an increased risk for the development of Parkinson’s disease in men who consumed 10 to 19.9 g of alcohol per day and in women who consumed 10 to 14.9 g of alcohol per day [42]. The study also revealed that heavy alcohol consumption, defined as having at least two drinks per day, was associated with an increased risk for Parkinson’s disease, while low to moderate alcohol consumption, i.e., beer drinking, less than one drink a day, was associated with a decreased incidence for the development of Parkinson’s disease.

Drinking more than the recommended limit of alcohol has also been shown to increase a person’s risk of developing Alzheimer’s disease [42]. Alzheimer’s disease is caused by a concentrated deposition of amyloid-β (Aβ) protein in the brain, memory failure, and dementia [43]). Preventative measures have encouraged low consumption of alcohol. Studies have reported that moderate consumption of ethanol may protect against the buildup of Aβ protein, though this advice is dangerous because “low to moderate” are poorly defined. The recommended maximum limits of alcohol, as mentioned previously, are reported to be 14 units each week for men and women, spread over three or more days.

Studies have found that a greater intake of alcohol is associated with a higher risk of dementia, which is marked by a decline in cognitive function, severe enough to interfere with daily life [43]. However, light to moderate consumption of alcohol can reduce one’s chances of developing dementia by 25–28%. The most common form of alcohol-related brain damage in persons with AUD-associated dementia, known as alcohol-related dementia, clinically presents as Korsakoff’s syndrome, called Korsakoff’s psychosis. Korsakoff syndrome is a chronic memory disorder caused by severe deficiency of thiamine (vitamin B1). The most common cause of Korsakoff syndrome is alcohol misuse, but AIDS and poor nutrition can also be associated with it. Since thiamine (vitamin B1) helps brain cells produce energy, when thiamine levels fall too low, the brain cannot generate enough energy to function properly. An overwhelming 80% of persons with AUD are vitamin B1-deficient.

The cerebrum controls mental processes such as memory, movement, and sensory perception. According to the 2nd edition of the Dictionary of Nursing [44], thiamine deficiency can result in confusion and memory loss. This is associated with Wernicke-Korsakoff syndrome (WKS), which is a disease that includes two separate syndromes: (1) Wernicke’s encephalopathy, which is considered the acute phase with a shorter duration and more serious symptoms, and (2) Korsakoff’s psychosis, a long-lasting and chronic condition that psychologically and socially debilitates the patient.

Korsakoff’s psychosis has been shown to be clearly associated with thiamine deficiency and is defined by severe retrograde and anterograde amnesia, meaning that the patient loses old memories and lacks an ability to form new ones [45]. In addition to thiamine deficiency, cortical atrophy caused by alcohol intoxication can cause frontal lobe dysfunction which worsens the effects of this condition [45]. It is important to note that the duration of a history of drinking plays a more significant role in the manifestation of Korsakoff’s psychosis as opposed to the quantity of drinking. People who have been drinking for long periods experience worse effects than those who have consumed significant amounts over a short period.

The pathological lesion associated with Wernicke’s encephalopathy is also located in the cerebrum. The encephalopathy results in extreme mental confusion and forgetfulness. Someone suffering from this component of WKS may have extreme difficulty doing something as common as exiting a room because of the disruption in brain functions, making it virtually impossible to comprehend the idea of a door or of exiting. Treatment is recommended in early stages of WKS and consists of administering vitamin B1. Patients that are unable to reverse their condition during later stages require custodial care and mental support. An article on “Alcoholic Brain Damage about Alcohol Research and Health” states that 25% of cases of WKS develop to a point where custodial care becomes necessary [46].

People who consume excessive amounts of alcohol may not walk properly, demonstrate poor coordination, and have impaired balance [47]. This is due to the effects of alcohol on the cerebellum. Like the cerebrum, the cerebellum is sensitive to excessive alcohol, and its malfunction is also associated with the psychosis characteristic of WKS. About 80% to 90% of persons with AUD who develop Wernicke’s encephalopathy also develop Korsakoff’s psychosis [45]. Similar to persons with AUD with encephalopathy, patients in this state are confused and have learning and memory problems. The difference, however, is that the psychosis often comes with physical debilitation, as it affects the cerebellum which controls the movements of the body. Although long-term abuse of alcohol is related to the development of the WKS or related dementias, light to moderate alcohol intake has been suggested to reduce the risk of dementia and Alzheimer’s disease [48]. A population-based prospective study done in Bordeaux, France, found that, for subjects drinking three to four standard glasses of wine per day, categorized as moderate drinkers, the odds ratio was 0.18 for incident dementia and 0.25 for Alzheimer’s disease. After adjusting for age, sex, education, occupation, baseline cognitive performances, and other possible confounders, the odds ratio was, respectively, 0.19 and 0.28. In the 922 mild drinkers (one to two glasses per day) there was a negative association only with Alzheimer’s disease after adjustment. The inverse relationship between moderate wine drinking and the incidence of dementia was explained neither by known predictors of dementia nor by medical, psychological, or socio-familial factors. Light-to-moderate drinking (one to three drinks per day) was significantly associated with a lower risk of dementia.

Alcohol is known to behave as a depressant in the central nervous system [20], thereby affecting a person’s ability to behave appropriately in response to environmental stimuli. Alcohol can affect the frontal lobe of the brain, which can make an individual act without thinking and makes it difficult to control the emotions. The frontal lobe, thalamus, and middle cerebellar peduncle have been demonstrated to be more vulnerable to the effects of acute alcohol consumption [49]. The frontal lobe is responsible for decision-making, planning, learning, and self-control. Gamma Aminobutyric Acid (GABA) is the brain’s primary inhibitory neurotransmitter that acts to slow neuronal signals along pathways in the brain, producing a natural calming effect [50]. When alcohol is present, the activity of the GABA receptors increases. This inhibits neuronal signals longer and allows the GABA receptor to produce its effects more extensively than in the absence of alcohol.

Alcoholism can alter the cognitive process. Some of the effects are memory loss, difficulty with learning, and emotional disturbances. Neuroimaging studies show that acute alcohol administration affects brain structures implicated in motivation and behavior control, and chronic intoxication is correlated with structural and functional abnormalities [51]. The cerebral cortex, which is responsible for thinking, senses, and the ability to make good judgments or to think clearly, is often impaired by alcohol.

When affected by alcohol, the hippocampus, which controls memory, can experience blackouts, possibly followed by memory loss. The hypothalamus coordinates important activities in the pituitary gland and autonomic nervous system and controls body temperature, hunger, thirst, and other homeostatic systems. After drinking too much alcohol, hunger, thirst, blood pressure, and the urge to urinate increase, while body temperature decreases.

The medulla oblongata is also affected by AUD [40]. The medulla is responsible for involuntary functions, such as body temperature regulation and breathing. When the medulla is affected, the body temperature drops, and respiration can be depressed.

### 2.9. Drugs: Effects of Alcohol

Consuming alcohol with other drugs whether simultaneously or sequentially can have a tragic effect. According to a Consumer Health News article on “Opioids and Alcohol, a Dangerous Cocktail”, when alcohol is consumed with opiates or “painkillers”, such as oxycodone, severe respiratory depression can occur [52]. In one study, 12 young volunteers aged between 21 and 28 years and 12 older volunteers aged between 66 and 77 years were observed as they consumed alcohol and opioids. None of the volunteers had taken opioids before. The study found that the consumption of only one oxycodone tablet with a “modest” volume of alcohol was sufficient to increase the risk of respiratory depression. The older individuals were more susceptible to the effects. Likewise, when taken with sedatives, alcohol, which is a depressant, works along with the sedative to slow the user’s heart rate and breathing. Because of alcohol’s damaging effects on the central nervous system, especially the parts of the brain that impact mood and behavior, when alcohol is taken with sedatives or antidepressants, there is a worsening of the behavioral and mental side effects. As a result, depression worsens and becomes harder to treat.

## 3. Epidemiology of Alcohol Abuse: Psychological and Societal Effects

In addition to the pathophysiological systemic and cellular effects, people suffering from alcoholism may also experience psychological and social effects. Social effects on mothers with AUD have been reported. According to the British Medical Journal, it was estimated that one every 67 women who consume alcohol during pregnancy will deliver a child with Fetal Alcohol Syndrome (FAS), which translates to about 119,000 children born with FAS in the world every year [53].

Psychological effects of alcohol can include severe depression, suicidal thoughts and tendencies, anxiety, violence, and unexplained mood swings. Persons afflicted by alcoholism often suffer from isolation and separation from their family, loved ones, and friends. Persons with AUD tend to socialize with individuals sharing a similar addiction. Alcoholism, therefore, not only poses physical and physiological effects, but also is often associated with negative psychological and social outcomes.

As children become young adults, many attend college away from home and use the extended freedom to engage in social drinking. “Drinking games”, or competitions in which individuals perform difficult tasks involving alcohol as either a punishment for losing or a reward for winning, encourage partygoers to drink more than they otherwise would. This can result in college atmospheres being especially dangerous for people prone to addiction and for persons with AUD behavior. The implication is that college environments can increase the risk of alcohol abuse and contribute to the problems related to it. Similar to many chronic diseases, alcoholism develops over time. Early symptoms include stress, which is associated with the availability of alcohol, a sudden gravitation in social groups that actively encourage drinking, and a reliance on drinking to achieve a specific psychological state [54]. Typically, the individual will begin to exhibit a heightened tolerance to alcohol, consuming more and noticeably showing less of the effects of alcohol consumption. In addition, a person’s ability to manage his/her drinking will decline. Persons with AUD are most often unaware of how much they drink.

## 4. Conclusions

Despite beneficial effects having been reported for mild alcohol consumption, the potential for negative life-threatening effects on multiple organs remains. The beneficial effects of alcohol appear to be dependent on multiple factors, including age, gender, co-morbidities, as well as the frequency and the amount of alcohol consumed [55]. The benefits of moderate alcohol consumption include a reduction in the risk of developing coronary heart disease, congestive heart failure, intermittent claudication, and myocardial infarction. The lack of clear guidelines on the amount and frequency of alcohol intake complicated by personal demographics make extrapolations to real-life practices, at best, difficult. Further study is needed on the effects of alcohol not only from a health perspective, but also from a societal standpoint.
